# Desorption of Coffee Pulp Used as an Adsorbent Material for Cr(III and VI) Ions in Synthetic Wastewater: A Preliminary Study

**DOI:** 10.3390/molecules27072170

**Published:** 2022-03-27

**Authors:** Dora Luz Gómez-Aguilar, Javier Andrés Esteban-Muñoz, Juan Pablo Rodríguez-Miranda, Deisy Baracaldo-Guzmán, Octavio José Salcedo-Parra

**Affiliations:** 1 Departamento de Química, Universidad Pedagógica Nacional, Bogotá 110231, Colombia or jestebanm@unal.edu.co (J.A.E.-M.); dbaracaldo@pedagogica.edu.co (D.B.-G.); 2 Facultad del Medio Ambiente y Recursos Naturales, Universidad Distrital Francisco José de Caldas, Bogotá 110231, Colombia; jprodriguezm@udistrital.edu.co; 3 Facultad de Ingeniería, Universidad Distrital Francisco José de Caldas, Universidad Nacional de Colombia, Bogotá 110231, Colombia; ojsalcedop@unal.edu.co or

**Keywords:** chromium, coffee pulp, desorption, preliminary study, synthetic wastewater

## Abstract

Some of the diverse agro-industrial waste generated in primary or secondary stages have proved to be promising biomaterials for treating aqueous effluents contaminated, in this case, with heavy metals. Therefore, it is necessary to know their optimal operating conditions and the regeneration or reusability of the solid by-product, an aspect related to desorption. Considering the above, this article presents the findings of a preliminary study related to the desorption process of coffee pulp without physicochemical modification (Castilla variety), an agricultural waste used as a sorbent of Cr(III and VI) ions in synthetic wastewater. The desorption efficiency of four eluting agents at defined concentrations (0.10M)—HC1, HNO_3_, H_2_SO_4_, and EDTA—was evaluated in a time interval of 1 to 9 days. Likewise, the proposals for the sorption and/or desorption mechanisms proposed and reported in the literature with respect to the use of biosorbents derived from the coffee crop are presented. With respect to the results, the coffee pulp used in previous studies of the adsorption of chromium species mentioned (optimal conditions in synthetic water of particle size 180 μm, dose 20 g·L^−1^, agitation 100 RPM, room temperature, time of 90 to 105 min) showed efficiencies in the removal of Cr(III) and Cr(VI) of 93.26% and 74.80%, respectively. Regarding the extracting substances used, H_2_SO_4_ 0.10 M was the one that presented the highest desorption percentage in both chromic species, with a desorption of 45.75% Cr(VI) and 66.84% Cr(III) in periods of 5 and 9 days, respectively, with agitation of 100 RPM and room temperature. Finally, the dissemination of preliminary results on the desorption of coffee pulp contaminated with chromic species without physicochemical modification is novel in this study, as similar work with this specific material has not yet been reported in the literature. On the other hand, the limitations of the study and future research are related to the evaluation at different concentrations and of other extractor solutions that allow improving the efficiency of desorption of these chemical species in a shorter time from the coffee pulp (with and without modification) as well as the reuse cycles. As a result, the desorption of coffee pulp used as an adsorbent material in real water could help researchers identify the possible interfering factors that affect the process (foreign anions and cations, organic matter, environmental conditions, among others).

## 1. Introduction

Water is one of the natural resources that is used in the processes of the diverse industrial activities in which it is required. This is due to the increasing industrialization that different countries in the world are going through. In order to safeguard public and environmental health, the aim is to ensure that these aqueous matrices are purified before their final disposal due to the significant negative effects they could have on the ecosystem in the short, medium, and long term [1,2].

Heavy metals (also known as environmentally priority pollutants, Potentially Toxic Elements (PTE), Persistent Bio-accumulative Toxics (PBT), and Unitary Potentially Toxic Residues (UPTR)) [3,4,5], are among the pollutants, produced by industrial wastewater, particularly those of an inorganic nature. These pollutants are not removed in conventional wastewater treatment plants (pretreatment, primary, secondary, and tertiary treatment stages) [6]. However, these substances can be mitigated through the use of advanced methodologies, which involve ion exchange, membrane filtration (microfiltration, nanofiltration, ultrafiltration, and reverse osmosis), electrodialysis, photocatalysis, flotation, and electrocoagulation, among others [7]. There are also conventional methods such as chemical precipitation and adsorption using activated carbon (AC) as a “universal adsorbent” and carbon nanotubes (CNTs) (since they are adsorbents that have high efficiency for both organic and inorganic pollutants removal) [3]. Although the advantages of these technologies refer to their high removal efficiency, generation of moderate amounts of sludge, and economy (exclusively the chemical precipitation); the disadvantages concern their high cost in implementation, requirements for energy, maintenance, and high cost of production, especially those of adsorption methods (AC) that prevent their use in countries with scarce resources [8,9,10].

In view of the above, over the years, research on so-called non-conventional technologies (also known as clean technologies or green methodologies) has gained considerable attention and importance. These include the use of biosorption, bioremediation, phytoremediation, hydrogels, and fly ash. This is mainly characterized by: (a) the use of live and dead biomasses, lignocellulosic materials, in order to revalue the various solid by-products generated in human activities susceptible to being reused in issues such as water treatment, and (2) being easy to acquire, highly efficient, environmentally friendly, high adsorption capacity, does not generate sludge and is economical [8,9,10,11].

Particularly for the biosorption method, important findings have been reported in the literature regarding the use of sorbents derived from agro-industrial by-products (coffee bagasse, coffee pulp, agave, maguey, sugar cane, organic waste from fruits and vegetables) [2,5,12]; solid materials from tannery industrial activities (chrome shavings, leather trimmings, fly ash, and hair) [13,14]; by-products of the incineration of municipal sewage sludge, such as fly ash and slag (chitosan, waste-to-energy power plant, sunflower wood, pine nut shells) [15,16,17,18]. Additionally, fruit and vegetable waste from the processing of the food industry, as well as the use of γ-polyglutamic acid, elderberries, pine biomass, and mushroom biomass [19,20,21,22]. These are proposed as possible alternatives to address the environmental challenges caused by various metal ionic species in aqueous effluents, such as Cr(III and VI), Ni(II), Zn(II), Pb(II), Cd(II), Mn(II), Cu(II), etc.

Biosorption is a physical method in which the adsorbates, pollutants in ionic state present in the liquid phase (wastewater), interact with the adsorbent (biological material) the solid phase in which the sorbates accumulate [23]. In this process, the physisorption and/or chemisorption processes are immersed; for the first, the types of binding forces are Van der Waals, with relatively low sorption heat (10 kJ·mol^−1^–40 kJ·mol^−1^), very low activation energy, and is fully reversible (desorption of the adsorbate occurs due to the decrease in the activity of the adsorbent on the fluid surrounding the surface). With respect to the second, the type of binding force is similar to chemical bonding, with high sorption heat (20 kJ·mol^−1^–400 kJ·mol^−1^), high activation energy, where the process is irreversible (the “desorbed compounds” are different from the adsorbed ones) [24].

Likewise, it is important to highlight that the opposite phenomenon of biosorption is desorption; in this process, the aim is to separate and/or recover the organic and inorganic pollutants that have interacted with the solid phase—biosorbent—with the purpose of reincorporating the substances into the industrial production processes, as well as the reuse of the sorbent material [25]. Publications on this topic have proposed the use of various extracting solutions or eluting agents, such as chelating agents (EDTA), acid substances (HCl, H_2_SO_4_), alkaline substances (NaOH), inorganic and organic salts (NaNO_3_, Ca(NO_3_)_2_, sodium citrate, etc.) or tests such as that reported by Ahalya et al. (2010) [26] that involve physisorption (physical bonding) and/or chemisorption (chemical bonding), exposing that if the physisorption process is present, the weakly bound metal ion can be easily desorbed with distilled water in most cases, but if the process is chemisorption or ion exchange or combination of the two, desorption can occur by stronger desorbing agents such as acidic or alkaline solutions, thus when using distilled water (pH 6.90) to desorb Cr(VI) from coffee husk, chemisorption was observed, since it was not desorbed with this type of water. Thus, the use of these substances will depend on variables such as: the type of sorbent, the metal ionic species present in the aqueous solution, pH, the concentration of the extractant solutions, contact time, temperature, ionic strength, agitation [8,25].

It is important to emphasize that desorption allows the recovery of the biomass free of contaminants to be reused in the biosorption process, thus allowing its regeneration. This is favorable because, in this way, it will be more accessible to users who implement this technology and with it, the maximum number of cycles in which the biosorbent can be reused is established. On the other hand, it is relevant to say that if organic and inorganic contaminants remain in solution, they can be reincorporated into the production cycle or life cycle of industrial processes, allowing companies to incorporate a circular economy and at the same time being sustainable, since they will favor environmental, economic, and social factors [2,5,7,26]. 

On the other hand, Cr(III) is one of the most stable chromium species found in high abundance in the environment [27,28] and it is considered an essential trace element for humans, causing the body to metabolize carbohydrates, proteins, and fats. Cr(VI) is also 1000 times more toxic than Cr(III), it causes allergies and skin ulcers, irritations on respiratory and gastrointestinal tract surfaces, fibrosis, alteration of genetic material, and immune system weakness. At the same time, renal and hepatic lesions have been observed in the long term after inhalation exposure. This has been linked to lung cancer according to the International Agency for Research on Cancer (IARC), the United States Environmental Protection Agency (USEPA), and as a probable carcinogen, based on epidemiological studies [29,30,31].

It is important to note tha the World Health Organization (WHO), ranks this heavy metal as one of the 13 with the greatest impact on public health and the environment [32] and that the United States Environmental Protection Agency (USEPA) ranks Cr(VI) as one of the 129 most critical pollutants [26]. Furthermore, chromium is used in the electroplating industry, tannery industry, dyes, electroplating, and other industries [33,34]. Some conventional methods for the treatment of chemical species such as Cr(VI) include reduction to Cr(III), then precipitation; additionally, electrolytic techniques, ion exchange, and other techniques are also used. [8]. Some of the maximum permissible limits for the two ionic species of chromium (III and VI) and total chromium in wastewater discharges in some countries reported in mg·L^−1^ are as follows: Colombia, México (Cr III and VI/ no apply), Cr Total 1.0–1.5; Venezuela ((Cr III/ no apply), Cr(VI) 0.5 and Cr Total 2.0) [28].

Regarding the evaluation of agro-industrial by-products in the context of industrial wastewater treatment, which incorporates pollutants such as the aforementioned, coffee pulp (CP) has been investigated in previous studies [2,5,27]. This lignocellulosic material generated from the pulping process represents 29% dry-weight of the coffee fruit; 2 tons of this residue are generated for each ton of coffee [35]. Every year, 21 billion kilograms of CP waste are produced worldwide, obtained as a by-product of the processing operations to obtain coffee beans [36]. Pulping and transportation of pulp with water, in addition to its disposal in uncovered or open pits, are considered to be responsible for 74% of the polluting potential represented by coffee by-products [37].

The most relevant coffee species on the planet is Arabica coffee, which constitutes 60% of world production due to its high quality [38]. Colombia is the third world producer after Brazil and Vietnam, with an approximate production of 14.30 million bags of 65 kg of coffee, while Brazil had a production of 63.40 million bags of 65 kg of coffee in 2020. Therefore, Brazil represents approximately 40% of world coffee production [39]. Regarding the almond coffee that Colombia exports in a year, approximately 162.900 tons of pulp residues are generated for every million 60 kg bags, which represents about 43.58% of the weight of the fresh fruit. If such tons of waste are not adequately treated, pollutants equivalent to the feces and urine of a population of approximately 868.736 inhabitants could be produced [36].

In terms of chemical composition, CP is composed of cellulose (18.65–65.50%), hemicellulose (0.98–3.30%), and lignin (12.20–19.70%) [35]. These compounds mainly have in their chemical structure aldehyde, methoxyl, phenol, ether, alcohol, aromatic rings, and carbonyl groups, which will be present in the various chemical interactions that may occur in the adsorption/desorption mechanisms between the sorbate and the adsorbent—depending on the conditions of the biosorption process (pH, temperature, dosage, etc.). In addition, CP has been given applications in animal feed, composting, biofuel production, production of energy drinks, production of coffee honey, vermicomposting, and larvicomposting [40,41].

Given the above, the present article shows the results of a preliminary study related to the desorption process of CP without physicochemical modification, with the material being used as a sorbent of ionic Cr(III and VI) species in synthetic wastewater. The use of four eluting agents (0.10M), HCl, HNO_3_, H_2_SO_4_, and EDTA was evaluated to desorb Cr species from CP. Likewise, the adsorption and desorption mechanisms proposed and reported in the research related to the use of sorbents derived from coffee cultivation and processing are presented. Finally, the authors present the limitations of the study and future research from this perspective. 

**Figure 1:**
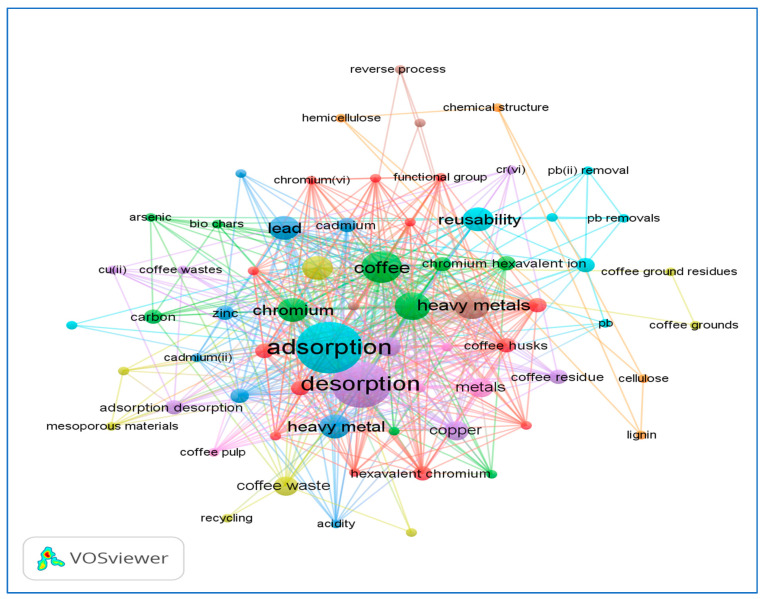
Bibliometric map related to co-occurrence of terms against the search from 2000 to the present for “desorption” and “coffee” and “heavy metals” (elaborated with Scopus metadata using VOSviewer 1.6.18 Software).

This research arises due to the low research related to this aspect, and that in publications related to agro-industry by-products, particularly derived from coffee cultivation, it has been raised as a future research and/or limitations, especially with this agricultural waste [5,27,42]. After a survey on sources of information from 2000 to 2021 using as a starting point the metadata of the Scopus database, a bibliometric map was made (see Figure 1) based on the co-occurrence of terms related to desorption, coffee pulp, and heavy metals. The above, with the use of the VOSviewer 1.6.18 software (free version), allowed the observation of a general panorama in terms of the research related to the aspect that is the subject of this article.

Figure 1 shows the co-occurrence of terms referring to the most mentioned words by the researchers and the relationships between them (e.g., adsorption, desorption, coffee beans, coffee leaves, heavy metals—copper, lead, chromium, cadmium, arsenic). This provides a first approach to the current research related to the desorption of lignocellulosic materials derived from the process of cultivation and processing of coffee fruit [5], except for CP contaminated with ionic Cr species.

## 2. Materials and Methods

### 2.1. Collection and Physical Treatment of CP

CP used was of the Castilla variety, collected from a coffee farm in the Maracas, Quebrada Negra, municipality of Neira, Department of Caldas (Colombia) with the coordinates 5.140579° N–75.484538° W. The sampling of the material was performed in zigzag, collecting a sample size of 10 kg that was dried for 7 to 15 days in solar parabolic dryers. This was to partially dehydrate the residue—the percentage of moisture obtained in this drying process was 8.20%. Subsequently, it was placed in an oven at 60 °C until it obtained a constant mass. Once the sample was dry, it was mechanically triturated in a mill to obtain a particle size of 180 µm [27,43].

### 2.2. Quantification of Cr(III and VI)

The Cr(III and VI) solutions were prepared using analytical reagents KCr(SO_4_)_2_ 99% and K_2_Cr_2_O_7_ 99.5%, which were diluted in deionized water at room temperature, from which stock solutions of 1000 mg·L^−1^ and diluted (1,5,10,15) mg·L^−1^ were prepared. The respective instrumental calibration was performed by the Atomic Absorption Spectrophotometry (AAS) method in a VARIAN AA 140 equipment; the method used was 3111 B of the Standard Methods using flame (air-acetylene).

### 2.3. Adsorption and Desorption Procedures

In previous studies, optimal biosorption conditions were found for each metal ionic species in volumes of 25 mL of synthetic wastewater (indicating that this water is prepared at a specific concentration) [44]. These corresponded to a mass of 0.50 g of CP, temperature of 20 °C, agitation of 100 RPM, and contact time of 90 and 105 min for Cr(III and VI), respectively. Furthermore, the pH values were 2.0 for Cr(VI) and 5.0 for Cr(III).

Subsequently, the CP contaminated with Cr(III and VI) ionic species was retained using Whatman grade 4 qualitative filter paper with a pore size of 20 µm–25 µm. Afterward, 0.10 g of the contaminated dry residue was immersed in a volume of eluting agent of 50mL each of HCl, HNO_3_, H_2_SO_4_, EDTA; all of concentration 0.10 M. The sorbent–eluent system was left under constant agitation at 100 RPM for 9 days at room temperature [12,45]. 

The extractive solutions were prepared from analytical grade reagents whose purities were HCl 37%, HNO_3_ 65%, H_2_SO_4_ 98%, EDTA 99%. In relation to the treatment of the data, the accumulated desorption percentage for each ionic species was plotted against the monitored time; additionally, the desorption capacity (Q_des._) was calculated by applying the following expression [30]:

Q_des_. (mg·g^−1^) = (C_des_·V)/M

C_des_: desorption concentration of the pollutant (mg·L^−1^).

V: volume (L) of the eluting agent.

M: mass (g) of the adsorbent.

Additionally:

%desorption = (C_des_/C_ads._) × 100

C_des._: desorption concentration in the extractant solution (mg·L^−1^).

C_ads._: sorption concentration of the biosorbent (mg·L^−1^).

### 2.4. Statistical Treatment

The Excel program’s ANOVA was used to perform the statistical analysis based on the comparison of desorption percentages and Cr(III and VI) ionic species with different extractant solutions.

## 3. Results and Discussion

### 3.1. Desorption Assays of Cr(III and VI) from CP

According to the preliminary adsorption studies, under the optimum conditions of pH, particle size, agitation, temperature, and adsorbent dosage, CP was used to remove Cr(III and VI) in synthetic wastewater. The optimum pH variables for Cr(III and VI) were 2.0 and 5.0 pH units, an initial concentration of 100 mg·L^−1^, a particle size of 180 µm, a temperature of 20 °C, and an adsorbent dose of 0.50 g with 25 mL of the synthetic solution for the two ionic species.

**Figure 2:**
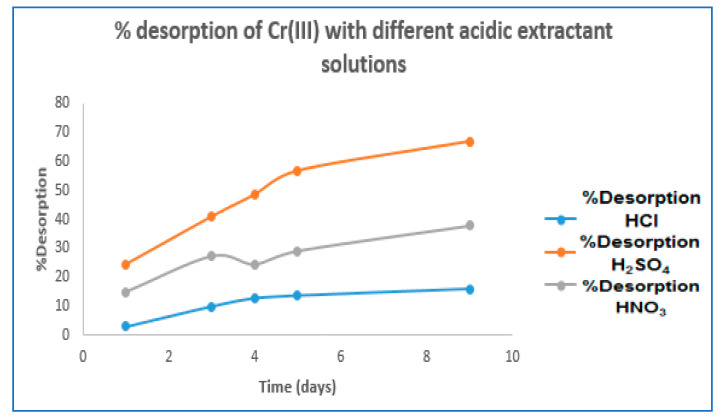
Percentage desorption of Cr(III) with different acidic extractant solutions.

Regarding Cr(III) desorption, it was performed using H_2_SO_4_, HCl, and HNO_3_ extractive solutions at the concentrations mentioned in the methodological section. The results are shown in Table 1 and Figure 2:

According to the results shown in Table 1 and Figure 2 H_2_SO_4_ 0.10 M demonstrated the highest Cr(III) desorption efficiency of 66.84% in a time of 9 days; similarly, the desorption capacities ranged for HCl at (0.23 mg·g^−1^–15.88 mg·g^−1^), H_2_SO_4_ (1.77 mg·g^−1^–4.85 mg·g^−1^), and HNO_3_ (4.72 mg·g^−1^–11.93 mg·g^−1^).

With the results, researchers used an ANOVA (*p* < 0.05) to specify if there were significant differences between the extractant solutions at the same concentration, temperature, agitation, and biosorbent material (see Table 2).

Table 2 shows a comparison of the desorption percentages for Cr(III) between the extractant solutions (HCl, H_2_SO_4_, and HNO_3_), showing that there are significant differences at 95% probability, given that the F calculated value is higher than the F critical value. Thus, it could be inferred that the desorption processes are different for each acid. 

**Figure 3:**
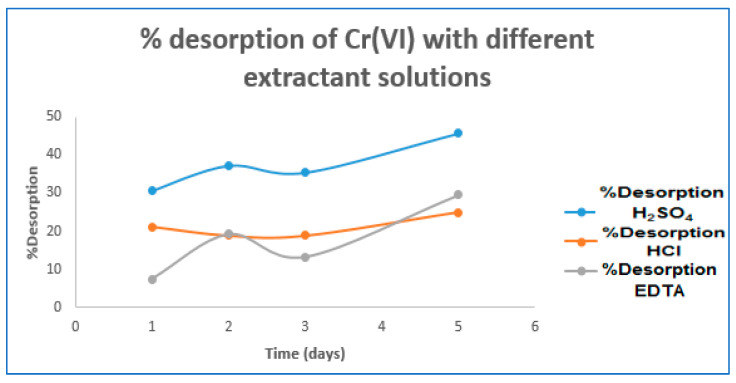
Percentage desorption of Cr(VI) with different extractant solutions.

On the other hand, Cr(VI) desorption was performed using H_2_SO_4_, HCl, and EDTA extracting solutions at the concentrations mentioned in the methodological section. The results are shown in Table 3 and Figure 3:

According to the results in Table 3 and Figure 2, it was found that of the three eluting agents evaluated, H_2_SO_4_ 0.10 M had the highest Cr(VI) desorption efficiency of 45.75% in a time of 5 days; similarly, the desorption capacities range for HCl was (1.44 mg·g^−1^–1.69 mg·g^−1^), for H_2_SO_4_ (2.08 mg·g^−1^–3.11 mg·g^−1^), and EDTA (1.21 mg·g^−1^–1.82 mg·g^−1^).

Having the results, researchers used an ANOVA (*p* < 0.05) to specify if there were significant differences between the extractant solutions at the same concentration, temperature, agitation, and biosorbent material (see Table 4).

Table 4 shows a comparison of the desorption percentages for Cr(VI) between the extractant solutions (H_2_SO_4_, HCl, EDTA); it can be observed that there are significant differences at 95% probability since the F calculated value is higher than the F critical value. Thus, it could be inferred that the desorption processes are different for each substance.

In the two desorption methods using CP as a sorbent, it was observed that a higher percentage of desorption occurred using acidic solutions since it is possible that the protons from the dissociation of the latter replace the metal (ion exchange) that is bound through electrostatic interactions to the functional groups on the surface of the adsorbent, for example, hydroxyls and carboxyls [25].

### 3.2. Reports on Mechanisms of Adsorption and Desorption of Agricultural Waste Derived from the Cultivation and Processing of Coffee

Table 5 shows the statements found and reported on the adsorption and desorption conditions using various by-products derived from coffee cultivation:

**Table 5:** Sorption and desorption processes and conditions with various by-products derived from coffee crops.

Ref.	By-Product of Coffee Cultivation and Desorbed Ionic Adsorbate	Adsorption Process and Conditions	Desorption Process	Conclusion of Desorption and/or Extractant/Eluent Solution Process, %Desorption
[11]	Coffee waste: “Greek coffee” drinks (Untreated and chemically treated—2% formaldehyde-) Cr(VI)	(a) Synthetic waters. (b) Volume of water: 50 mL. (c) Sorbent dosage: 1 g·L^−1^. (d) pH: 5.0. (e) Particle size: 475 μm–525 μm. (f) Agitation: 140 RPM. (g) Pollutant concentration: 50 mg/L. (h) Temperature: 25 °C. (i) Optimum time: 24 h. (j) Maximum sorption capacity: 38.68 mg·g^−1^–43.75 mg·g^−1^. (k) %Efficiency: 60–62.	(a) Synthetic waters. (b) Volume of water: 50 mL. (c) pH adjustment: 2.0–12.0 units. (d) Extraction solution: NaOH 0.10 M and HCl 0.10 M. (e) Volume of eluting agent: Not reported. (f) Particle size: 475 μm–525 μm. (g) Sorbent dosage: 1 g·L^−1^. (h) Agitation: 140 RPM. (i) Temperature: 25 °C. (j) Desorption time: 1440 min.	Acidic conditions favored desorption (optimum desorption pH 2.0); (%desorption: 84–95).
[26]	Coffee husk Cr(VI)	(a) Synthetic water. (b) Volume of water: 100 mL. (c) Sorbent dosage: 1 g·L^−1^. (d) pH: 2.0. (e) Particle size: Not reported. (f) Agitation: Not reported. (g) Pollutant concentration: 10 mg·L^−1^–100 mg·L^−1^. (h) Temperature: 25 °C. (i) Optimum time: 24 h. (j) Maximum sorption capacity: 43.75 mg·g^−1^. (k) %Efficiency: 60–62.	(a) Synthetic water. (b) Volume of water: 100 mL. (c) pH adjustment: Not reported. (d) Extraction solution: NaOH 0.02 M (e) Volume of eluting agent: Not reported. (f) Particle size: Not reported. (g) Sorbent dosage: 1 g·L^−1^. (h) Agitation: Not reported. (i) Temperature: 25 °C. (j) Desorption time: 180 min.	Desorption with NaOH 0.02 M; (%desorption: 60).
[8]	Exhausted ground coffee wastes Cr(VI)	(a) Synthetic waters. (b) Volume of water: 100 mL. (c) Sorbent dosage: 6.67 g·L^−1^. (d) pH: 3.0. (e) Particle size: 0.75 mm–1.50 mm. (f) Agitation: 30 RPM. (g) Pollutant concentration: 50 mg/L. (h) Temperature: 20 °C ± 2 °C. (i) Optimum time: 120 min. (j) Maximum sorption capacity: 10.17 mg·g^−1^. (k) %Efficiency: Not reported.	(a) Synthetic water. (b) Volume of water: Not reported. (c) pH adjustment: Not reported. (d) Extraction solution: NaOH and HCl (in the range of 0.01 M to 1 M). (e) Volume of eluting agent: 0.1 g of sorbent in 15 mL. (f) Particle size: Not reported. (g) Sorbent dosage: Metal loaded on biosorbent (10 mg Cr/g sorbent dry) (h) Agitation: Not reported. (i) Temperature: 25 °C. (j) Desorption time: 24 h.	Solution NaOH 1 M better for desorbing total Cr from the sorbent (Cr III and VI species); (%desorption: 47).
[30]	Coffee ground Cr(VI)	(a) Synthetic waters. (b) Volume of water: 25 mL. (c) Sorbent dosage: 2 g·L^−1^. (d) pH: 2.0. (e) Particle size: Not reported. (f) Agitation: 250 RPM. (g) Pollutant concentration: 10 mg·L^−1^–30 mg·L^−1^. (h) Temperature: 30 °C. (i) Optimum time: 180 min. (j) Maximum sorption capacity: 87.72 mg·g^−1^. (k) %Efficiency: Not reported.	(a) Synthetic waters. (b) Volume of water: Not reported. (c) pH adjustment: 10.0 (d) Extraction solution: NaOH 0.1 M (e) Volume of eluting agent: Not reported. (f) Particle size: Not reported. (g) Sorbent dosage: Not reported. (h) Agitation: 150 RPM. (i) Temperature: 25 °C. (j) Desorption time: 60 min.	NaOH 0.1 M (pH: 10); (%desorption: 10-15).
[29]	Coffee husk Ash Cr(VI)	(a) Synthetic water. (b) Volume of water: 100 mL. (c) Sorbent dosage: 1.5 g·L^−1^. (d) pH: 2.0. (e) Particle size: Not reported. (f) Agitation: 100 RPM. (g) Pollutant concentration: 0.5 mg/L. (h) Temperature: 22 °C ± 2 °C. (i) Optimum time: 40 min. (j) Maximum sorption capacity: 15.53 mg·g^−1^. (k) %Efficiency: >90%.	(a) Synthetic water. (b) Volume of water: Not reported. (c) pH adjustment: Not reported. (d) Extraction solution: NaOH (range 0.01 M to 0.5 M). (e) Volume of eluting agent: 100 mL. (f) Particle size: Not reported. (g) Sorbent dosage: Not reported. (h) Agitation: Not reported. (i) Temperature: 25 °C. (j) Desorption time: Not reported.	NaOH (range 0.01 M to 0.5 M; (%desorption: 77.33).
[46]	Coffee leaves Variety Castillo Cr(VI)	(a) Synthetic water. (b) Volume of water: 50 mL. (c) Sorbent dosage: 2 g·L^−1^. (d) pH: 4.0. (e) Particle size: 0.149 mm (f) Agitation: 0 RPM. (g) Pollutant concentration: 1000 mg·L^−1^. (h) Temperature: 25 °C. (i) Optimum time: 60 min. (j) Maximum sorption capacity: Not reported. (k) %Efficiency: 82.	(a) Synthetic water. (b) Volume of water: Not reported. (c) pH adjustment: Not reported. (d) Extraction solution: HCl 0.1 M and H_2_SO_4_ 0.1 M. (e) Volume of eluting agent: 10 mL. (f) Particle size: Not reported. (g) Sorbent dosage: Not reported. (h) Agitation: without agitation. (i) Temperature: 25 °C. (j) Desorption time: 60 min.	The strong acids used were not very effective in desorbing the metal ionic species; however, 0.1 M HCl showed a higher desorption grade of 25% in a volume of 10 mL.
[27]	Coffee pulp Cr(VI)	(a) Synthetic waters. (b) Volume of water: 50 mL. (c) Sorbent dosage: 20 g·L^−1^. (d) pH: 2.0. (e) Particle size: 0.18 mm. (f) Agitation: 100 RPM. (g) Pollutant concentration: 20 mg·L^−1^–500 mg·L^−1^. (h) Temperature: 25 °C. (i) Optimum time: 105 min. (j) Maximum sorption capacity: 13.48 mg·g^−1^. (k) %Efficiency: 74.80.	Conditions found in this study about desorption. (a) Synthetic waters. (b) Volume of water: Does not apply. (c) pH adjustment: Does not apply. (d) Extraction solution: HCl 0.1 M, H_2_SO_4_ 0.1 M, EDTA 0.1 M. (e) Volume of eluting agent: 50 mL. (f) Particle size: 0.18 mm. (g) Sorbent dosage: 0.1 g/50 mL. (h) Agitation: 100 RPM. (i) Temperature: 20 °C. (j) Desorption time: 5 days.	The desorption percentage was 45.75% in a time of 5 days using H_2_SO_4_ 0.1 M in this study.
This study	Coffee pulp Cr(III)	(a) Synthetic waters. (b) Volume of water: 50mL. (c) Sorbent dosage: 20 g·L^−1^. (d) pH: 5.0. (e) Particle size: 0.18 mm (f) Agitation: 100 RPM. (g) Pollutant concentration: 20 mg·L^−1^–500 mg·L^−1^. (h) Temperature: 25 °C. (i) Optimum time: 90 min. (j) Maximum sorption capacity: 7.41 mg·g^−1^. (k) %Efficiency: 93.26.	(a) Synthetic waters. (b) Volume of water: Does not apply. (c) pH adjustment: Does not apply. (d) Extraction solution: HCl 0.1 M, H_2_SO_4_ 0.1 M, HNO_3_ 0.1 M. (e) Volume of eluting agent: 50 mL. (f) Particle size: 0.18 mm. (g) Sorbent dosage: 0.1 g/50 mL. (h) Agitation: 100 RPM (i) Temperature: 20 °C. (j) Desorption time: 9 days.	The desorption percentage was 66.84% in a time of 9 days using H_2_SO_4_ 0.1 M.

When analyzing Table 5 in relation to the variables (particle size, contact time, adsorbent dosage, and temperature) used in the desorption process using coffee wastes (leaves, beans, and pulp), it was observed that the extracting solutions used were inorganic acids (HCl, H_2_SO_4_), as well as basic solutions such as NaOH in concentrations ranging from 0.02 M to 1 M. Furthermore, it can be established that the basic solutions have been one of the most used eluting agents, where their desorption percentages have ranged from 10% to 77.3%, while with the acids, the most efficient has been HCl 0.1 M, where the desorption percentages ranged from 25% to 95% given that the contact times were between 60 and 1440 min, indicating that the longer the time, the higher the desorption percentage. In relation to the reports found and shown in the table above, it is observed that the ionic species that has been adsorbed and desorbed with the coffee residues has been only Cr(VI) and the matrix used was synthetic wastewater.

The results obtained in the present study establish adsorption/desorption data with two ionic species, Cr(III) and Cr(VI), where the acidic extractive solutions (HNO_3_ 0.1 M and H_2_SO_4_ 0.1 M) were more efficient in the desorption of the two species with CP. Additionally, the Q_des_. of each species were reported, which were 11.93 mg·g^−1^ and 3.11 mg·g^−1^ for Cr(III) and Cr(VI) with extractive solutions such as HNO_3_ and H_2_SO_4_, respectively.

**Figure 4:**
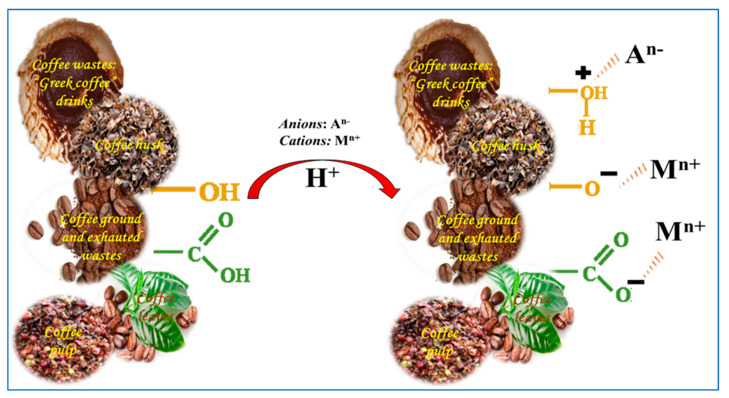
Mechanisms of adsorption of anionic and cationic species using sorbents derived from coffee cultivation and processing.

**Figure 5:**
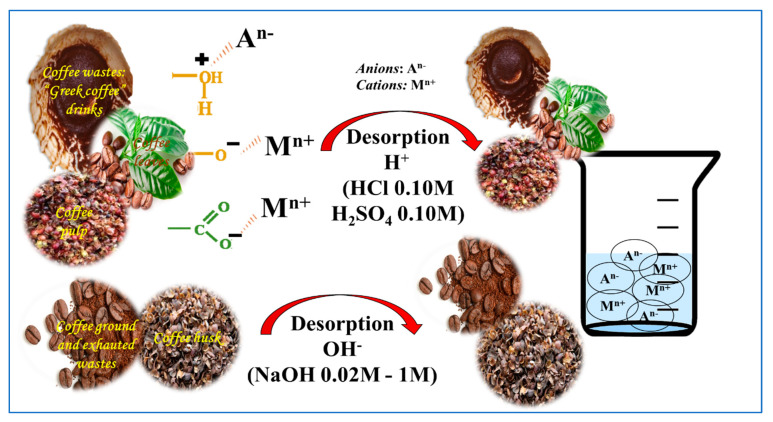
Desorption mechanisms of anionic and cationic species using sorbents derived from coffee cultivation and processing.

In addition to the above, Table 6, Figure 4 and Figure 5 show and illustrate the statements reported in the literature related to the mechanisms of adsorption and desorption when using the residues from coffee cultivation and processing.

### 3.3. Limitations and Future Research

According to the preliminary study and the findings presented in this article, it is important to mention that:

(a) Subsequent studies are required to evaluate additional extracting solutions, in addition, to those evaluated here, in order to improve the percentage desorption of CP without physicochemical modification. Furthermore, it is relevant to analyze the effects of the eluting agents’ concentrations.
(b) It is necessary to carry out adsorption and desorption processes (reuse cycles) in order to analyze and evaluate the number of times that CP can be used under the same conditions.
(c) Studies related to the application of the sorbent in real water and/or multimetallic aqueous systems are still lacking, which would allow analyzing the effect of other pollutants present as well as atmospheric conditions that could intervene in the sorption and desorption of CP being used as a sorbent for Cr ions. Likewise, physicochemical modification of the biosorbent is necessary in order to observe the increase and/or decrease in sorption tests in the future.
(d) Analyses focused on the environmental impact and techno-economic studies related to the adsorption and desorption of lignocellulosic materials such as CP are required in order to analyze the short, medium, and long term effects of scaling up the technology in small, medium, or large-scale treatment systems, which involve environmental, economic, and social aspects.
(e) It is recommended to carry out a triplicate for each concentration used in the extractive solutions, in order to make a statistical treatment based on the repeatability and reproducibility of the desorption method to be used on a pilot and/or industrial scale with real water.

## 4. Conclusions

In previous studies, it was established that coffee pulp, without physicochemical modification, had Cr(III) and Cr(VI) removal efficiencies in synthetic wastewater of 93.26% and 74.80%, respectively. The above, under optimum conditions of 20g/L dosage, pH 5.0 and 2.0, time of 90 min and 105 min, respectively, and particle size of 180 μm.

Four extractive solutions were evaluated to desorb chromium ionic species (Cr III and VI) from coffee pulp without physicochemical modification (Castilla variety); these corresponded to HCl, HNO_3_, H_2_SO_4_, and EDTA, all at concentrations of 0.10 M. It was established that sulfuric acid was the best agent to elute these sorbates under agitation conditions of 100 RPM, room temperature, for periods of 5 and 9 days, with desorption percentages of 45.75% and 66.84%, respectively. Additionally, the Q_des._ of each species were reported, which were 11.93 mg·g^−1^ and 3.11 mg·g^−1^ for Cr(III) and Cr(VI) with extracting solutions such as HNO_3_ and H_2_SO_4,_ respectively.

In addition, according to the review of the literature, there are few studies related to the desorption of materials derived from coffee cultivation, some of which are related to coffee waste, coffee husks, and coffee grounds. Regarding the mechanisms of adsorption and desorption, they are mainly related to electrostatic interactions, oxidation–reduction reactions, and ionic exchange. This is related to the physicochemical composition of the materials, where the OH and COOH groups of compounds such as lignin, cellulose, and hemicellulose predominate. 

Since no reports related to CP desorption have yet been published in the literature, these preliminary findings will be used as starting points for future studies wishing to investigate the use of CP as a heavy metal sorbent in real wastewater at the pilot and/or industrial scale.

## Figures and Tables

**Table 1:** Percentage desorption of Cr(III) and Q_des._ with different acidic extractant solutions.

Time (days)	%Desorption HCl	HCl Q_des._ (mg·g^−1^)	%Desorption H_2_SO_4_	H_2_SO_4_ Q_des._ (mg·g^−1^)	%Desorption HNO_3_	HNO_3_ Q_des._ (mg·g^−1^)
1	3.16	0.23	24.43	1.77	14.94	4.72
3	9.97	0.72	40.89	2.97	27.32	8.62
4	12.72	0.92	48.49	3.52	24.40	7.70
5	13.61	0.99	56.72	4.12	29.06	9.17
9	15.88	1.15	66.84	4.85	37.80	11.93

**Table 2:** Analysis of variance between extractant solutions for Cr(III).

%Desorption HCl	%Desorption H_2_SO_4_	%Desorption HNO_3_	F Calculated	F Critical *p* < 0.05
3.16	24.43	14.94	29.98	4.26
9.97	40.89	27.32		
12.72	48.49	24.40		
13.61	56.72	29.06		
15.88	66.84	37.80		

**Table 3:** Percentage desorption of Cr(VI) and Q_des_. with different extractant solutions.

Time (days)	%Desorption H_2_SO_4_	H_2_SO_4_ Q_des._ (mg·g^−1^)	%Desorption HCl	HCl Q_des._ (mg·g^−1^)	%Desorption EDTA	EDTA Q_des._ (mg·g^−1^)
1	30.56	2.08	21.17	1.44	7.39	1.21
2	37.24	2.53	18.87	1.28	19.21	1.48
3	35.42	2.41	18.87	1.28	13.21	1.41
5	45.75	3.11	24.91	1.69	29.58	1.82

**Table 4:** Analysis of variance between extractant solutions for Cr(VI).

%Desorption H_2_SO_4_	%Desorption HCl	%Desorption EDTA	F Calculated	F Critical *p* < 0.05
30.56	21.17	7.39	9.43	5.14
37.24	18.87	19.21		
35.42	18.87	13.21		
45.75	24.91	29.58		

**Table 6:** Statements reported on possible adsorption and desorption mechanisms using coffee by-products as sorbents.

Ref.	By-Product of Coffee Cultivation	Explanation of the Adsorption and/or Desorption Mechanism
[11]	Coffee waste: “Greek coffee” drinks (Untreated and chemically treated—2% formaldehyde)	Since the optimum pH (5.0) found in the study was higher than the pHzcp (3.4), it was established that the surface of the biosorbent is negative. In the case of the Cu(II) species, it is favorable from the point of view of the electrostatic interactions that could occur (dissociation of the COOH group, which predominates in the material, followed by the phenolic and lactonic groups). However, in the case of the Cr(VI) species, being anionic species such as (Cr_2_O_7_)^2−^, (CrO_4_)^2−^, (HCrO_4_)^−^, they could bind to the basic functional groups present (since in the sorbent there is coexistence with the acid groups). Likewise, the OH group coming from the lignin and cellulose compounds could be positively charged, forming the oxonium ion (due to the high concentration and high mobility of H^+^), favoring the interactions with the anionic species of chromium, which are related to the desorption process involving the use of acid (ion exchange).
[26]	Coffee husk	It is established that the predominant functional groups in the material are COOH and OH. Due to the acidic conditions of the system, the Cr(VI) species present are (Cr_2_O_7_)^2−^, (CrO_4_)^2−^, (HCrO_4_)^−^ ions; likewise, it is established that under acidic conditions, the surface of the sorbent begins to protonate and attract these anionic species, an assumption that is related to the formation of the oxonium ion, originating from electrostatic interactions. In relation to the alkaline desorption of metallic ionic species, the process of chemisorption or ion exchange is proposed. It is established that at high pH, the OH ions would release the chromic ions from the sorbent following an ion exchange mechanism.
[8]	Exhausted ground coffee wastes	Since the optimum pH (3.0) found in the study was lower than the pHzcp (3.90), it was established that the surface of the biosorbent is positive; the chromic ionic species present at the pH worked (1.0–9.0) were (CrO_4_)^2−^, (HCrO_4_)^−^ and Cr. Given this, electrostatic interactions could be present there. On the other hand, it was established that according to the literature, at acid pH Cr(VI) can probably be reduced to Cr(III) after contact with the lignocellulosic waste; this is because the reduction of the Cr(VI) species “consumes” protons from the medium, which is favored at low pH, establishing that the coffee beans would have the “ability” to reduce Cr^6+^ to Cr^3+^ (favored at pH: 1.0). As a result, the Cr(III) species released into the aqueous medium would be present as Cr^3+^ and Cr(OH)^2+^ cations, which could not be attracted by the positive charges on the adsorbent surface. Given this, the present mechanism involves oxidation–reduction reactions (involving the H^+^ present in the medium, the probable conversion of some alcohol groups of the sorbent into carboxylic functions), and electrostatic interactions.
[30]	Coffee ground	It is expressed that the functional groups that mainly compose the material are the OH, NH, and CH groups coming from the compounds of lignin, cellulose, hemicellulose, and protein. At strongly acidic pH, the (HCrO_4_)^−^ species predominates, and it is easily bound to the positive charges of the biosorbent through electrostatic interactions. However, at higher pH a competition of (CrO_4_)^2−^ and OH ionic species is generated, interfering with the binding sites on the sorbent. Thus, desorption is a reversible process and occurs by the action of the eluting agent NaOH.
[29]	Coffee husk Ash	Given the strongly acidic conditions (pH: 2.0), it is established that redox reactions could occur, in which the Cr(VI) species is reduced to Cr(III), through the mechanisms illustrated by the chemical equations below: (Cr_2_O_7_)^2−^_(ac)_ + 14H^+^_(ac)_ + 6e^−^ →2Cr^3+^_(ac)_ + 7H_2_O_(ac)_ (HCrO_4_)^−^_(ac)_ + 7H^+^_(ac)_ + 3e^−^ → Cr^3+^_(ac)_ + 4H_2_O_(ac)_ Likewise, it is stated that the diffusion of (Cr_2_O_7_)^2−^ and (HCrO_4_)^−^ ions neutralize the negative ionic charges due to the high mobility and concentration of H^+^ ions. The decrease of the chromate sorption process with the increase of the pH of the solution could be due to the negative surface charge of the biosorbent and the surface adjustment with OH ions that could cause repulsions between the OH ions and the chromate ion. Therefore, the pH value must be decreased because doing so would increase the positive charge on the coffee husk ash due to the protonation process.
[46]	Coffee leaves Variety Castillo	The total chromium biosorption process was favored under highly acidic conditions, which could be explained by the chemical equilibrium presented between the chromate–dichromate species, as shown by the chemical equation: 2(CrO_4_)^2−^_(ac)_ + 2H^+^_(ac)_ ⇌ (Cr_2_O_7_)^2−^_(ac)_ + H_2_O_(ac)_ It is noted that decreasing the pH would generate an increase in the concentration of H^+^ ions. This would produce a displacement of the equilibrium towards the formation of the (Cr_2_O_7_)^2−^ species (a more stable species in the aqueous medium).
[27]	Coffee pulp	It was established that the sorbent presents a predominance in the composition of lignin and cellulose, compounds that are quite important given that the functional groups that compose them interact under optimal conditions in terms of Cr(VI) biosorption; in relation to the functional groups, -OH and -COOH are highlighted. On the other hand, the optimum pH found in the study (2.0) for Cr(VI) was lower than the pHzcp (3.95), indicating that the sorbent surface is positively charged. In addition, it was found that the total active sites of acidic character predominate over the basic ones, while for Cr(III) the optimum pH was 5.0, in this case, the adsorbent surface is negative. The possible sorption mechanism that occurs between the sorbent and the ionic sorbate in the aqueous solution (chromic species (HCrO_4_)^−^, (Cr_2_O_7_)^2−^, (CrO_4_)^2−^) is related to electrostatic interactions. This is because the OH groups coming from the lignin and cellulose structures are protonated, forming the oxonium ion (this is due to the highly acidic conditions of the system).

## Data Availability

Not applicable.

## Sample Availability

Not available.
